# Differences in Colorectal Cancer Outcomes by Race and Insurance

**DOI:** 10.3390/ijerph13010048

**Published:** 2015-12-22

**Authors:** Rima Tawk, Adrian Abner, Alicestine Ashford, Clyde Perry Brown

**Affiliations:** 1 Institute of Public Health, College of Pharmacy and Pharmaceutical Sciences, Florida A&M University, Tallahassee, FL 32307, USA; 2 Residential Services Administrator, Marianna, FL 32448, USA; a_abner21@yahoo.com (A.A.); alicestine.ashford@famu.edu (A.A.); perry.brown@famu.edu (C.P.B.)

**Keywords:** colorectal health, access to care, health disparities, social determinants of health

## Abstract

Colorectal cancer (CRC) is the second most common cancer among African American women and the third most common cancer for African American men. The mortality rate from CRC is highest among African Americans compared to any other racial or ethnic group. Much of the disparity in mortality is likely due to diagnosis at later stages of the disease, which could result from unequal access to screening. The purpose of this study is to determine the impact of race and insurance status on CRC outcomes among CRC patients. Data were drawn from the Surveillance, Epidemiology, and End Results database. Logistic regressions models were used to examine the odds of receiving treatment after adjusting for insurance, race, and other variables. Cox proportional hazard models were used to measure the risk of CRC death after adjusting for sociodemographic and tumor characteristics when associating race and insurance with CRC-related death. Blacks were diagnosed at more advanced stages of disease than whites and had an increased risk of death from both colon and rectal cancers. Lacking insurance was associated with an increase in CRC related-deaths. Findings from this study could help profile and target patients with the greatest disparities in CRC health outcomes.

## 1. Introduction 

Colorectal cancer (CRC) is the second most common cancer among African American women and the third most common cancer for African American men. Health disparities in CRC treatment and outcomes have been widely documented for several decades. CRC disproportionately affects Blacks in the US who have higher incidence and mortality compared with Whites [1,2,3]. Much of the disparity in mortality is likely due to diagnosis at later stages of the disease [4,5,6,7,8], which could result from unequal access to screening [9]. These racial and ethnic disparities in outcomes have been attributed to a wide range of potential factors, with socioeconomic status (SES) at the forefront [10]. SES could serve not only as a proxy for income, education, and access to care and usage of healthcare services, but also as a surrogate of health risk factors and behavior [11]. While SES may be a determinant of survival, there may be a complex interaction between SES and race and ethnicity. In fact, the causes of these inequalities are complex and are thought to reflect social and economic disparities more than biologic differences associated with race. These include inequities in work, wealth, income, education, housing and overall standard of living, as well as barriers to high-quality cancer prevention, early detection, and treatment services [12]. Much of the difference in survival is believed to be due to barriers that prevent timely and high-quality medical care, which results in later stage at diagnosis, as well as disparities in treatment. In fact, about half of the reductions in incidence and mortality have been attributed to expanded use of effective screening tests to identify and treat high-risk adenomas and early stage cancers [12]. 

Furthermore, Blacks tend to develop colorectal cancer at an earlier age, with a higher prevalence of right-sided neoplasia [13,14,15]. These findings prompted the American College of Gastroenterology to recommend that Blacks be screened for colorectal cancer beginning at 45 years of age (rather than the widely accepted 50 years of age for average-risk persons) and that colonoscopy be used as the preferred screening method [13]. Some studies highlighted that racial disparities in CRC rates and outcomes may be due more to differences in health-care utilization than biological differences [16]. Other studies, however, have shown that even with a similar distribution of tumor stage by race or after statistical adjustment for stage, survival disparities between African-Americans and Caucasians persist [17]. 

Several studies have evaluated racial trends in CRC survival using SEER program data [1,5]. The impact of health insurance on the treatments and outcomes in patients with CRC has not been well documented using SEER dataset. To our knowledge, this is the first study to investigate the impact of insurance and race on CRC outcomes using SEER dataset. Our current study compensates for the limitations of prior research since the previous study was restricted to a single year [18]. The purpose of this study is to determine the impact of race and insurance on CRC outcomes (treatment and survival) in CRC patients from 2007 to 2010 after adjusting for sociodemographic and tumor characteristics.

## 2. Ethics Statement

The proposal for this study was reviewed and approved by the Institutional Review Board of the Florida A &M University as an exempt study (protocol#: 013-84). 

## 3. Methods

In this study, we used data from the National Cancer Institute’s Surveillance, Epidemiology, and End Results (SEER). SEER database is the most widely used and recognized source of US estimates of cancer prevalence, incidence, mortality, and survival and includes patient demographics, and cancer type and stage. We analyzed data from 18 population-based cancer registries included in the SEER program of the National Cancer Institute. The study population consisted of residents who were diagnosed with colon or rectal cancer during the period 2007–2010 and who were living in state or county-based registries. Follow-up information is also collected for patients in the registry. The termination date for entry and follow-up was 31 December 2010. To be included in the study, participants had to experience the diagnosis of colon or rectal cancer as the first lifetime cancer diagnosis. Patients with a single primary diagnosis of cancer of the colon or rectum (International Classification of Diseases for Oncology, 3rd edition site codes: C180 to C189 for colon while C199 and C209 for rectum) were selected. 

SEER data included information on patient demographics (*i.e.*, age at diagnosis, sex, race/ethnicity, marital status, primary payer of health insurance). For insurance, indicator variables were as follows: Medicaid, Medicare, private, and no health insurance. Tumor characteristics and treatment consisted of site of cancer (colon vs rectum), tumor stage, tumor grade, surgery, and radiation. Stage was defined at the time of diagnosis by the SEER Site-Specific Summary Staging Guide and was classified as in situ, local, regional, or distant. Cause specific survival (time from CRC diagnosis to death due to CRC) was the major outcome measure. Patients who died due to other reasons during study period or were alive at end of study were statistically censored. 

Logistic regressions models were used to examine the adjusted associations of late-stage CRC diagnosis with insurance coverage and race. The dependent variable was the stage of diagnosis (late *vs.* early), and the independent variables were individual patient characteristics (race, age, insurance, marital status). Multivariate relationships between individual patient characteristics (race, age, insurance, marital status) and the odds of receiving surgical or radiation treatments were examined by multiple logistic regressions. Surgery is the main treatment for colon cancer whereas surgery and radiation are used more commonly for rectal cancer. The determinants of treatment and survival were examined separately for colon and rectal cancers. For cancer-specific mortality, the adjusted risk of death using Cox proportional hazards regression analysis was examined after controlling for socio-demographics, (age, gender, race, insurance, marital status) and tumor characteristics (stage and grade). Statistical analyses were performed using SAS Version 9.4 (LOGISTIC, LIFETEST, and PHREG procedures).

## 4. Results

We studied a total of 32,339 patients including 23,456 with colon cancer and 8,883 with rectal cancer. Descriptive statistics were used to summarize patient characteristics (Table 1). Study population was older (80.33% more than 50 years old), had more whites, 53.29% were married and included a greater proportion of patients on Medicare coverage (53%). Blacks had 18% higher odds of late-stage CRC diagnosis (OR, 1.181; 95% CI 1.103–1.264) as compared to whites. Uninsured patients had 65% increased odds of late-stage CRC diagnosis than privately insured patients (Table 2). Multivariate determinants of receiving treatment for colon and rectal cancer are presented in Table 3, Table 4 and Table 5. Race and insurance were not significant predictors of receipt of treatment for both colon and rectal cancers. There were no racial differences in the use of radiation therapy.

Only patients who were covered by Medicare had about 30% decreased odds of receipt of radiation therapy (*p* < 0.0001) for rectal cancer. The adjusted risk of colon cancer death was 9% higher for African Americans as compared to whites, 48% percent higher for uninsured patients and 59% percent higher for Medicaid patients than privately insured patients (Table 6). The adjusted risk of rectal cancer-related death was about 20% higher for African Americans than whites, 62% percent higher for uninsured patients and 36% percent higher for Medicaid patients than privately insured patients (Table 7). Hispanics were included in the adjusted analysis but the estimates were not shown in both Table 6 and Table 7 since they were not significant for both colon and rectal cancers (*p* = 0.2648 and *p* = 0.846, respectively). In addition, age was accounted for in the analysis but was not reported since it was not significant except for those aged 71 years and above. 

**Table 1:** Characteristics of the study population (N = 32,339).

Variable		N	Percent
Gender	Male	16,598	51.3
	Female	15,741	48.7
Age at diagnosis	18–50	4349	13.4
	51–70	14,381	44.5
	71–80	7413	22.9
	>80	6196	19.2
Race	Non-Hispanic Whites	25,979	80.3
	Non-Hispanic Blacks	4032	12.5
	Hispanics	2328	7.2
Marital Status	Married	17,232	53.3
	Single	4712	14.6
	Other	10,395	32.1
Insurance	Uninsured	709	2.2
	Medicaid	2426	7.5
	Private	12,069	37.3
	Medicare	17,135	53.0
Anatomic location of cancer	Colon	23,456	72.5
	Rectum	8883	27.5
Tumor stage	In situ	1963	6.0
	Stage I	7566	23.0
	Stage II	7945	24.6
	Stage III	8251	26.0
	Stage IV	6614	20.4
Diagnosis stage	In situ	1086	3.3
	Localized	12,375	38.3
	Regional	11,971	37.0
	Distant	6907	21.4
Tumor Grade	Well-differentiated	2721	8.41
	Moderately differentiated	18,987	58.7
	Poorly differentiated	5215	16.1
	Undifferentiated	644	2.0
	Unknown	4772	14.8
Stage late	Yes	14,865	46.0
	No	17,474	54.0
Surgery	No	32,095	99.2
	Yes	244	0.8
Radiation	No	27,820	86.0
	Yes	4519	14.0

**Table 2:** Adjusted associations with late-stage colorectal cancer (CRC).

	Odds Radio	LCL	UCL	*p*-Value
Race				
Non-Hispanic Whites	**1.000**			
Non-Hispanic Blacks	1.181	1.103	1.264	<0.0001
Hispanics	1.089	0.999	1.186	0.0532
Insurance	****			
Private	**1.000**			
Medicaid	1.187	1.085	1.299	0.0002
Medicare	0.840	0.800	0.881	<0.0001
Uninsured	1.654	1.414	1.934	<0.0001
Marital Status				
Married	**1.000**			
Single	1.103	1.031	1.180	0.0042
Other	1.026	0.975	1.079	0.3233

**Table 3:** Adjusted associations with receipt of surgery for colon cancer.

	Odds Radio	LCL	UCL	*p*-Value
Race				
Non-Hispanic Whites	**1.000**			
Non-Hispanic Blacks	0.786	0.404	1.529	0.4790
Hispanics	0.425	0.132	1.369	0.1517
Insurance	****			
Private	**1.000**			
Medicaid	1.881	0.930	3.804	0.0788
Medicare	0.603	0.357	1.019	0.0586
Uninsured	2.225	0.767	6.459	0.1412
Marital Status				
Married	**1.000**			
Single	1.458	0.778	2.732	0.2389
Other	1.224	0.719	2.085	0.4567

**Table 4:** Adjusted associations with receipt of surgery for rectal cancer.

	Odds Radio	LCL	UCL	*p*-Value
Race				
Non-Hispanic Whites	**1.000**			
Non-Hispanic Blacks	0.966	0.574	1.627	0.8976
Hispanics	1.492	0.911	2.444	0.1117
Insurance	****			
Private	**1.000**			
Medicaid	1.225	0.704	2.133	0.4731
Medicare	1.338	0.958	1.868	0.0878
Uninsured	0.473	0.115	1.952	0.3005
Marital Status				
Married	**1.000**			
Single	1.553	1.027	2.346	0.0367
Other	1.109	0.770	1.595	0.5786

**Table 5:** Adjusted associations with receipt of radiation for rectal cancer.

	Odds Radio	LCL	UCL	*p*-Value
Race				
Non-Hispanic Whites	**1.000**			
Non-Hispanic Blacks	0.899	0.779	1.037	0.1425
Hispanics	1.045	0.892	1.224	0.5841
Insurance	****			
Private	**1.000**			
Medicaid	0.857	0.725	1.014	0.0714
Medicare	0.717	0.624	0.823	<0.0001
Uninsured	1.009	0.769	1.325	0.9471
Marital Status				
Married	**1.000**			
Single	0.956	0.845	1.081	0.4732
Other	0.906	0.819	1.003	0.0562

**Table 6:** Adjusted associations with colon cancer-related death.

Variable		HR (95% CI)	*p*-Value
Race	Blacks *vs.* Whites	1.097 (1.000, 1.193)	0.0326
Insurance	Medicaid *vs.* Private	1.594 (1.407, 1.806)	<0.0001
	Medicare *vs.* Private	1.286 (1.135, 1.455)	<0.0001
	Uninsured *vs.* Private	1.480 (1.219, 1.795)	<0.0001

**Table 7:** Adjusted associations with rectal cancer-related death.

Variable		HR (95% CI)	*p*-Value
Race	Blacks *vs.* Whites	1.198 (1.022, 1.405)	0.0258
Insurance	Medicaid *vs.* Private	1.360 (1.124, 1.646)	0.0016
	Medicare *vs.* Private	1.166 (0.953, 1.426)	0.1363
	Uninsured *vs.* Private	1.627 (1.233, 2.165)	0.0008

## 5. Discussion

African Americans had increased odds of late stage CRC as compared to whites. In addition, participants who were on medicaid or uninsured had increased odds of late stage diagnoses as compared to those on private insurance. Race was not associated with receipt of treatment for both colon and rectal cancers. We anticipated that uninsured participants would have decreased odds of receipt of treatment. However, this association failed to reach statistical significance. African Americans had increased risk of death from both colon and rectal cancers. Uninsured participants as well as those covered by Medicaid had increased risk of death as compared to those on private insurance. Our findings are consistent with the literature. Previous studies have shown that African Americans have a higher likelihood of being diagnosed with more advanced tumors as compared to whites [1,19,20].

For CRC, lack of insurance is associated with an elevated risk of late-stage diagnosis and a decreased likelihood of undergoing screening and receiving treatment following a diagnosis [2]. Our study failed to capture differences in receipt of CRC treatment between African Americans and whites even though the disparities in CRC treatment have been reported in the literature. Gross *et al.* in his study found out that black patients were less likely than white patients to receive therapy for cancers of the colon. Authors suggested that racial disparities in cancer care are not due to geographic variation in the patterns of care but are likely to be attributed to the differential impact of access to care, cost, and health beliefs or preferences [21].

For death related outcomes, our study found that African Americans had an increased risk of death due to colon cancer and a greater risk of death due to rectal cancer. Our findings are consistent with another study that found that African Americans experienced poorer survival than whites for colon or rectum cancers. However, authors found that improvements in survival for CRC were more pronounced for younger patients [22] which was not captured in our study since age estimates were not significant. Another study conducted by Silber *et al.* found that the persistent disparity in colon cancer survival seemed to be associated with presentation characteristics at diagnosis (demographic characteristics, comorbid conditions, and tumor characteristics including stage and grade) rather than treatment differences [23]. We found that the diagnosis stage was a major predictor for both colon and rectum cancers survival. 

Survival was worse for uninsured patients and those with Medicaid coverage than for privately insured patients for both colon and rectal cancers. To our knowledge, no other study has investigated the issue of survival according to race and insurance using SEER data for CRC.

Our study had some limitations. SEER database does not collect information on SES, education, income, lifestyle factors, comorbidities, use of screening tests, or receipt of chemotherapy. Another limitation is that insurance status assessed at the time of diagnosis may have changed over the follow-up period. Data from state tumor registries may not fully capture treatments given in outpatient settings and may not capture treatments received out of state. One potential limitation of an observational study is that unmeasured confounders or other unknown differences between black and white patients could impact CRC outcomes.

In spite of marked advances in CRC survival, it is increasingly important to make equal access to care a national priority and to identify the key obstacles to receipt of and benefit from treatment. Identifying factors associated with different improvement in cancer survival and understanding differences in the rates of improvement in survival among the different populations can inform future improvements in cancer care. 

## 6. Conclusions

Uninsured patients and those covered by Medicaid presented with more advanced disease than did privately insured patients. Findings from this study demonstrate that patients without insurance experience worse outcomes and would benefit the most from improved access to screening and optimal cancer care. Results could help profile and target patients with the greatest disparities in CRC health outcomes. Therefore, continued research efforts are necessary to disentangle the clinical, social, biological, and environmental factors that constitute the racial disparity. 
